# Investigating the Role of OrbF in Biofilm Biosynthesis and Regulation of Biofilm-Associated Genes in *Bacillus cereus* BC1

**DOI:** 10.3390/foods13050638

**Published:** 2024-02-20

**Authors:** Yang Sun, Wenjing Shuai, Lanmengya Nie, Xiangfei Li, Ling Jiang

**Affiliations:** 1 State Key Laboratory of Materials-Oriented Chemical Engineering, College of Food Science and Light Industry, Nanjing Tech University, Nanjing 211816, China; sunyang@njtech.edu.cn (Y.S.); 202361119008@njtech.edu.cn (W.S.); 13638176707@163.com (L.N.); jiangling@njtech.edu.cn (L.J.); 2 Engineering Laboratory for Industrial Microbiology Molecular Beeding of Anhui Province, College of Biologic & Food Engineering, Anhui Polytechnic University, 8 Middle Beijing Road, Wuhu 241000, China

**Keywords:** *Bacillus cereus*, transposon TnYLB-1, biofilm formation, regulator OrbF, regulatory mechanism

## Abstract

*Bacillus cereus* (*B. cereus*), a prevalent foodborne pathogen, constitutes a substantial risk to food safety due to its pronounced resilience under adverse environmental conditions such as elevated temperatures and ultraviolet radiation. This resilience can be attributed to its capacity for biofilm synthesis and sustained high viability. Our research aimed to elucidate the mechanisms governing biofilm biosynthesis in *B. cereus*. To this end, we constructed a 5088-mutant library of the *B. cereus* strain BC1 utilizing the transposon TnYLB-1. Systematic screening of this library yielded mutants exhibiting diminished biofilm formation capabilities. Twenty-four genes associated with the biofilm synthesis were identified by reverse PCR in these mutants, notably revealing a significant reduction in biofilm synthesis upon disruption of the *orbF* gene in *B. cereus* BC1. Comparative analysis between the wild type and *orbF*-deficient BC1 strains (BC1Δ*orbF*) indicated a marked downregulation (decreased by 11.7% to 96.7%) in the expression of genes implicated in biofilm formation, flagellar assembly, and bacterial chemotaxis in the BC1Δ*orbF*. Electrophoretic mobility shift assay (EMSA) further corroborated the role of OrbF, demonstrating its binding to the promoter region of the biofilm gene cluster, subsequently leading to the suppression of transcriptional activity of biofilm-associated genes in *B. cereus* BC1. Our findings underscore the pivotal role of *orbF* in biofilm biosynthesis in *B. cereus*, highlighting its potential as a target for strategies aimed at mitigating biofilm formation in this pathogen.

## 1. Introduction

Pathogenic microorganisms such as *Bacillus cereus*, *Escherichia coli*, and *Listeria monocytogenes* have extensively contaminated global food supplies, posing a significant threat to food safety and contributing to foodborne diseases [1]. According to estimates from the World Health Organization in 2021, approximately 600 million people worldwide suffer from foodborne diseases each year, resulting in around 420,000 deaths. *B. cereus* is implicated in 1.4% to 12% of all global food poisoning incidents [2]. A study conducted by the State Key Laboratory of Applied Microbiology Southern China revealed that about 27% of samples from pasteurized milk, even after undergoing pasteurization, were contaminated with *B. cereus* [3]. Statistics indicate that 80% of bacterial infections are associated with bacterial biofilms. Bacteria within biofilms exhibit altered morphology and physiological functions compared to free-living bacteria, leading to an increase in antibiotic tolerance by 10 to 1000 times [4]. Therefore, it is crucial to eradicate biofilms during the food production process to ensure the safety of both food and public health.

Due to the strong contaminating and pathogenic properties of *B. cereus*, current research is predominantly focused on the identification [5], epidemiology and molecular’ characteristics [6], virulence traits, and antibiotic resistance of *B. cereus* [7]. However, food spoilage is a complex process, and traceback efforts are often just the starting point. Therefore, there is an urgent need to understand the behavior of foodborne pathogenic bacteria at the molecular level [8]. The formation of biofilms is a primary mechanism by which foodborne microorganisms persist under pressure conditions during the food production process, representing a significant concern in the field of food safety. Biofilms are structured communities composed of bacterial cells from the same or different species, enhancing cell resistance to stressors associated with food (such as temperature changes, dryness, and pH variations) and antimicrobial agents. Biofilm formation regulatory networks in *B. subtilis* have been thoroughly investigated in previous studies [9,10]. Nevertheless, the regulatory mechanisms governing biofilm formation in *B. cereus* are currently poorly understood. Consequently, issues related to *B. cereus* biofilm formation, such as quorum sensing, transposon-mediated exploration of novel genes, and the elucidation of signaling pathways, are gradually becoming focal points in both domestic and international research [11,12,13].

**Figure 1:**
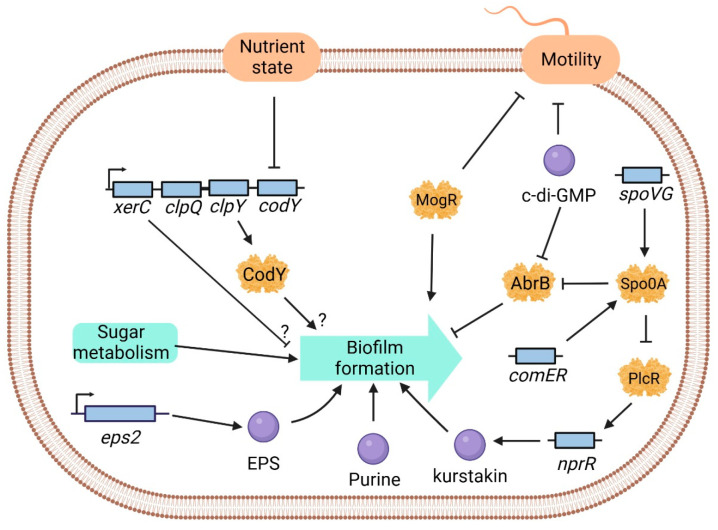
Schematic representation of the regulatory network controlling biofilm formation in *B. cereus*. Yellow proteins represent transcription factors that play pivotal roles in orchestrating the genetic regulation of biofilm formation, purple circles represent metabolites involved in the biofilm synthesis pathway, contributing to the intricate molecular processes, and light blue rectangles denote open reading frames (ORFs) associated with genes participating in biofilm formation. The question mark indicates that *clpY* affects biofilm formation through an unknown mechanism. (This figure has been modified based on the reference [19]).

*B. cereus* regulates the expression of a series of target genes through quorum sensing (QS) and its interaction with transcription factors. This regulation influences microbial behaviors such as biofilm formation, acid resistance, production of antimicrobial factors, and the control of virulence factor expression, contributing to food spoilage and bacterial pathogenicity issues [14,15]. Ding’s research group at Jinan University, through transposon random mutagenesis, discovered that the deletion of the *flgE* gene in *B. cereus* inhibits biofilm formation [16]. Slack et al. [17], by performing in-frame deletion of the *clpY* gene, enhanced biofilm formation. The *clpY* gene encodes the ATPase subunit of the ClpY-ClpQ protease complex, which is located in an operon with *clpQ*, *codY*, and *xerC* (Figure 1). Yan et al. [18] found that mutations leading to impaired biofilm formation are located in genes such as *comER*, *purD*, *purH*, *aad*, and *pepP*. According to functional predictions, these genes are associated with crucial processes such as nucleotide biosynthesis, iron salvage, antibiotic production, ATP-dependent proteolysis, and transcriptional regulation, indicating their vital role in biofilm formation. Functional analysis of *comER* revealed its positive regulatory role in both biofilm and spore formation, potentially through its impact on Spo0A activity, a global transcription factor for spore and biofilm formation in most rods (Figure 1). However, the identification of additional transcription factors related to *B. cereus* biofilm and the elucidation of the biofilm regulatory mechanisms remain crucial challenges that need urgent resolution.

To identify more novel genes related to designing biofilm synthesis in Bacillus cereus, we constructed mutant libraries using the TnYLB-1 transposon. The plasmid pMarA harbors a temperature-sensitive replication system, repG+^ts^, which allows replication at 30 °C but not at 50 °C. Under the conditions of 50 °C, the Himar I transposase on pMarA is expressed under the control of the PB promoter, enabling it to recognize the inverted repeat sequences (ITR) at both ends of the transposon TnYLB-1. This allows the transposon to undergo random transposition from the plasmid to the host bacterial genome, leading to its insertion into the host genome [20]. Therefore, in this study, *E. coli* DH5α/pMarA-TnYLB-1 was used as the donor strain, and *B. cereus* BC1 was used as the recipient strain to construct a *B. cereus* TnYLB-1 transposon insertion mutant library. Mutant strains with impaired biofilm synthesis were selected. Through reverse PCR identification of these selected *B. cereus* mutants, the disrupted genes were identified, revealing the involvement of the RNA polymerase sigma factor *SigB* encoding gene *J8Y18_05105*, as well as genes related to flagellum synthesis and secretion, including *flgG* [21], *flgE* [16], *motB* [22], the adenylyl cyclase encoding gene *J8Y18_20970*, and the TIGR04197 family type VII secretion effector encoding gene *J8Y18_14985*. Additionally, three previously uncharacterized transcriptional regulatory factor encoding genes *J8Y18_01585*, *J8Y18_16035*, and *J8Y18_09555* were identified. Building upon the identification of the *B. cereus* biofilm synthesis negatively correlated gene *orbF*, the molecular mechanisms underlying OrbF regulation of biofilm synthesis in *B. cereus* were preliminarily elucidated.

## 2. Materials and Methods

### 2.1. Bacterial Strains and Growth Conditions

*B. cereus* BC1 and its derivatives were cultured in tryptic soy broth (TSB) at 37 °C, 200 rpm, or on nutrient agar plates at 37 °C in standing culture. *E. coli* strains were grown in Luria–Bertani (LB) medium at 37 °C. The following concentrations of antibiotics were added when needed: 10 μg/mL erythromycin (Beijing Solarbio Science & Technology Co., Ltd., Beijing, China) 50 μg/mL kanamycin (Beijing Solarbio Science & Technology Co., Ltd., Beijing, China) for *B. cereus* growth, and 100 μg/mL ampicillin (Shanghai Boer Chemical Reagents Co., Ltd., Shanghai, China) for *E. coli* growth. The strains and plasmids used in this work are listed in Table S1. Primers are listed in Table S2.

### 2.2. Construction of Transposon Mutagenesis Library

Considering the varying temperature sensitivities of different bacteria, based on the principle of the pMarA-TnYLB-1 plasmid high-temperature-induced transposition technique, this study established different temperature gradients between 40 °C and 50 °C. Additionally, different treatment times were set for each temperature gradient to determine the optimal conditions for the highest transposition efficiency. Subsequently, under these identified optimal conditions, the TnYLB-1 transposon was induced to randomly insert into the BC1 genome. Transposition mutants were then screened and identified on kanamycin plates.

The pMarA-TnYLB-1 plasmid was introduced into the BC1 strain through electroporation, and resistance colonies against erythromycin and kanamycin were selected at 30 °C. The transformed BC1 strains were selected and cultured in LB liquid medium containing kanamycin (50 µg/mL) and erythromycin (10 µg/mL) at 30 °C with agitation at 200 rpm for 24 h. The bacterial cultures were then diluted to 10^−3^ and 10^−4^, and each dilution was plated on LB (kanamycin 50 µg/mL) agar plates. These plates were subjected to different temperature conditions and treated for various durations, creating the following treatment combinations: 40 °C, 42 °C, 44 °C, 46 °C, 48 °C, and 50 °C, each treated for 4 h, 6 h, 8 h, and 10 h to induce plasmid suicide. Subsequently, the plates were transferred to a 30 °C incubator for overnight cultivation.

A sterile toothpick was used to pick a single colony grown on LB (kanamycin 50 µg/mL) agar plates. Simultaneously, the picked colony was inoculated at corresponding positions on kanamycin (50 µg/mL) LB agar plates and erythromycin (10 µg/mL) LB agar plates. The cultures were then incubated overnight at 30 °C. Bacteria that grew on kanamycin (50 µg/mL) LB plates but not on erythromycin (10 µg/mL) LB plates were selected, indicating that mutants were resistant to kanamycin but sensitive to erythromycin. The biofilm phenotype of each mutant was screened using a 96-well microplate. DNA was extracted from mutants containing transposon insertions, and the DNA samples were subjected to PCR for detection. To determine the transposon insertion site, genomic DNA was digested with the restriction enzyme Taq I (Takara, Dalian, China). Self-ligation was performed with T4 DNA ligase at 25 °C for 10 min, followed by reverse polymerase chain reaction amplification of the TnYLB-1 transposon insertion sequence using oIPCR-F/oIPCR-R. After sequencing, the insertion site was identified using local BLAST.

### 2.3. Constructing In-Frame Knockout Strains

The mutants were constructed using the method according to Sun et al. [23], with slight modifications. For gene knockout, the upstream (containing 9 bp of the gene to be knocked out) and downstream (containing 20 bp of the gene to be knocked out) homology arms of the gene to be knocked out were amplified by PCR. The plasmid pHT304-TS [24] was linearized by double enzyme (*Eco*R I, *Hin*d III) digestion, and the linear DNA fragment obtained above was recombined using the ClonExpress Ultra One Step Cloning Kit V2 (Vazyme Biotech Co., Ltd., Nanjing, China). The recombinant plasmid pHT304-TS-*orbF* was transformed into *E. coli* DH5α competent cells, and positive clones were selected and used to verify successful construction.

The method was constructed following Li et al. with slight modifications [16]. To construct the *orbF* mutant, the obtained recombinant plasmid was transformed into *B. cereus* BC1. The transformation mixture was prepared by adding 1 mL SOC medium (2% (*w*/*v*) Tryptone, 0.5% (*w*/*v*) Yeast Extract, 0.05% (*w*/*v*) NaCl, 2.5 mM KCl 10 mM MgCl_2_, 20 mM glucose) to suspend the bacteria. The mixture was then cultured at 30 °C and 200 rpm for 3 h. After cultivation, the bacteria were centrifuged at 6000× *g* for 2 min at 30 °C. The pellet was plated on LB agar containing erythromycin, and the plates were incubated overnight at 30 °C. The potential transformants were confirmed by PCR. Positive transformants were transferred to culture medium containing erythromycin and incubated at 42 °C and 200 rpm for 6–12 h. This process was repeated six times. Subsequently, the strains were cultured at 30 °C and 200 rpm for 6–12 h, a total of 9–12 times. The bacterial liquid was plated on LB agar without antibiotics. Colonies were then transferred to LB agar plates containing antibiotics. Colonies that did not grow on the plates were subjected to PCR detection and sequencing. For the construction of the complementary strain, the pHT304 plasmid [25] was used. The gene was cloned into pHT304 using the ClonExpress Ultra One Step Cloning Kit V2 (Vazyme Biotech Co., Ltd., Nanjing, China) as described above. The recombinant plasmid was electroporated into the mutant, and experiments were conducted using positive transformants as described below.

### 2.4. Growth Curve of Different Strains

To analyze the growth curves of *B. cereus*, exponential-phase cells of BC1, BC1Δ*orbF*, and BC1Δ*orbF::orbF* (OD_600_ = 0.6) were inoculated at 3% inoculum into fresh TSB medium. The growth of *B. cereus* cells was determined by monitoring the optical density (OD) of the cultures at 600 nm every hour. The curve of OD_600_ nm versus incubation time was plotted.

### 2.5. Biofilm Formation Assay

For the analysis of biofilm formation, *B. cereus* strains were cultured overnight at 37 °C with agitation at 200 rpm. Subsequently, 2 microliters of the overnight culture were transferred into a 6-well plate with 2 mL of TSB medium in every well, followed by static incubation at 37 °C for a duration of 12 h. Biofilm determination was conducted following the protocols outlined in a previously reported work [26]. Bacteria from overnight cultures were diluted 50-fold using fresh TSB medium containing 0.5% glucose. The diluted cultures were added to a sterile 6-well plate and incubated at 37 °C for 24 h. Adherent bacteria were stained with a 0.1% crystal violet solution, and the remaining stain was gently rinsed with Phosphate-Buffered Saline (PBS, pH 7.4). The stained cells were lysed and dissolved with 33% acetic acid, and the absorbance was measured and quantified at a wavelength of 492 nm using a spectrophotometer.

### 2.6. Swimming and Swarming Assay

The method was constructed using the method according to Pan et al., with slight modifications [27]. For swarming and swimming assays, a volume of 1 μL of exponential-phase cells (OD_600_ of 0.6) from BC1, BC1Δ*orbF*, and BC1Δ*orbF::orbF* strains was spotted onto 0.5% and 0.3% semisolid LB media, respectively. The measurement of swarming and swimming zones was conducted after incubation at 37 °C for 16 h and 24 h, respectively.

### 2.7. RNA Extraction and Quantitative Real-Time PCR

The method was constructed following Sun et al. with slight modifications [28]. RNA was prepared by static cultivation in LB medium at 37 °C. In brief, total RNA was treated with RNase-free pipette tips, followed by purification using the FastPure Cell/Tissue Total RNA Isolation Kit (Vazyme Biotech Co., Ltd., Nanjing, China). Reverse transcription of 1 µg total RNA was performed using 4 µL of 5× HiScript II qRT SuperMix II (Vazyme Biotech Co., Ltd., Nanjing, China). The resulting cDNA was subjected to real-time quantitative PCR analysis using AceQ qPCR SYBR Green Master Mix (Vazyme Biotech Co., Ltd Nanjing, China), with primer details provided in Supplementary Table S1. The 16S rRNA was used as an internal reference gene, and real-time monitoring was conducted using the StepOnePlus PCR System (Applied Biosystems, Foster City, CA, USA).

### 2.8. OrbF Clone Expression, Preparation and Purification

The amplification primers (*orbF*-F/*orbF*-R) for the *orbF* gene were designed based on the *B. cereus* genome sequence in GenBank (accession number: CP072774.1). The *orbF* gene was amplified using genomic DNA from *B. cereus* BC1 as a template. The obtained target gene fragment was ligated to the linearized pET-28a vector using the ClonExpress Ultra One Step Cloning Kit V2. The ligated product was then transformed into *E. coli* BL21 competent cells, spread onto plates containing kanamycin (50 μg/mL), and incubated overnight. Positive transformants were selected and verified for the presence of the recombinant plasmid. The recombinant plasmid was sent to Shanghai Sangon Biotechnology Co., Ltd. for sequencing validation, and the resulting strain was named BL21/*orbF*.

The verified *E. coli* positive transformants were activated and then transferred to 50 mL LB medium containing kanamycin (50 μg/mL) at an inoculum of 1%, incubated at 37 °C and 180 rpm with shaking, cultured until the OD was about 0.6–0.8 when IPTG (final concentration of 0.5 mM) was added, and induced to express at 16 °C overnight. The above induced organisms were collected and washed three times with PBS (pH 7.4) buffer, and the cells were resuspended and sonicated under cooling conditions for 10 min to obtain the crude enzyme solution, which was then filtered through a 0.45 µm membrane, and finally, the target proteins were purified by using a Ni-NTA protein purification column, and the protein concentration was determined using a Bradford protein assay kit (Sangon Biotech, Shanghai, China).

### 2.9. Electrophoretic Mobility Shift Assay

The method was adapted from a previously published protocol with slight modifications [29]. As described in item 2.8 of this study, the OrbF protein was obtained. The promoter of the biofilm synthesis gene cluster was amplified and ligated to the plasmid pMD19T (simple), resulting in the construction of the recombinant plasmid pMD19T-Promoter. Using pMD19T-Promoter as a template, the promoter was amplified with fluorescence-labeled primers M13-47-Cy3 and RV-M-Cy3. The purified protein and Cy3-labeled promoter were incubated in 2× binding buffer (40 mM Tris-HCl, pH 7.5; 4 mM MgCl_2_; 100 mM NaCl; 10% glycerol; 2 mM DTT; 0.2 mg/mL BSA; 0.02 mg/mL poly(dI-dC); and 1 mM EDTA) at 25 °C for 30 min. The incubated mixture was electrophoresed on a 5% native gel for approximately 1 h or 1.5 h, and the results were visualized using the Image Quant LAS 4000 system.

### 2.10. Statistical Analysis

Experiments were conducted at least twice, with a minimum of three biological replicates. Error bars represent the standard deviations from three independent experiments and the Origin software (10.1.0.1780) was used to perform weighted regression, Student’s *t*-tests, and one-way analysis of variance with Tukey’s posttest. Significance was set at a *p* value of < 0.05 (***, *p* < 0.001; **, *p* < 0.01; *, *p* < 0.05).

## 3. Results and Discussion

### 3.1. Transposon Efficiency

Due to the temperature sensitivity of the pMarA plasmid, we designed six different temperature gradients and four different treatment times to obtain the optimal mutation temperatures and times of TnYLB-1 transposon. The experimental results showed that the transposition efficiency would increase with the increase in temperature, the optimal temperature was 46 °C, and above 46 °C, the transposition efficiency decreased significantly. The concomitant increase in treatment time also enhances the transposition efficiency, and the transformants were best treated at 46 °C for 10 h (Table 1).

The pMarA plasmid, under various combinations of treatment time and temperature conditions, facilitated the transposition of the TnYLB-1 transposon, inserting it into the genome of *Bacillus cereus* BC1. Due to the temperature sensitivity of the pMarA plasmid, it hinders plasmid replication under transposition temperatures. Therefore, after TnYLB-1 completed transposition, the plasmid backbone could not persist in the host bacteria, resulting in positive transformants resistant to kanamycin but sensitive to erythromycin. Single colonies that grew on kanamycin plates after treatment at 46 °C were selected using a sterilized toothpick and simultaneously streaked onto kanamycin (50 µg/mL) and erythromycin (10 µg/mL) plates. The cultures were then incubated overnight at 30 °C. Mutant strains showing resistance to kanamycin and sensitivity to erythromycin were screened and selected.

### 3.2. Screening of Key Genes for Biofilm Synthesis in Bacillus cereus

**Figure 2:**
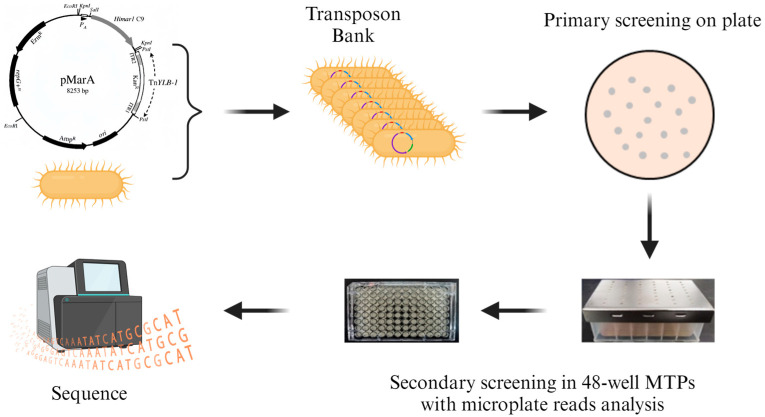
Workflow for screening biofilm formation defect mutants using the TnYLB-1 mutant library.

To screen genes associated with biofilm synthesis in *B. cereus*, we used *B. cereus* BC1 as the starting strain. Employing the method depicted in Figure 2, the donor strain *E. coli* DH5α/pMarA-TnYLB-1 was delivered into *B. cereus* BC1 through electroporation, creating a transposon insertion mutant library. Subsequently, 5088 *B. cereus* mutant strains were obtained through screening. These mutants were cultured in a 96-well plate for 3 days to assess their ability to form biofilms at the air–liquid interface and on the surface of polystyrene pegs. Mutants exhibiting growth similar to the wild type in planktonic culture but lacking biofilm formation after 3 days were designated as biofilm-deficient. Mutants resembling the wild type in planktonic culture but failing to adhere to peg surfaces after 24 h were identified as submerged biofilm-deficient. Further screening yielded 200 mutant strains, and their biofilm synthesis was observed through static cultivation in 24-well plates.

To analyze the reasons behind the low efficiency of biofilm synthesis in the mutant strains, this study conducted reverse PCR to identify the disrupted genes. Ultimately, the transposon TnYLB-1 was found to have inserted into 24 different genes or intergenic regions (Table 2). Based on previous literature, these genes were classified into three categories: (i) Disrupted genes at the insertion site were identified as genes related to flagellar synthesis and secretion, including *flgG* [21], *flgE* [16], *motB* [22], adenylate cyclase gene encoding a protein of the type II secretion system (*J8Y18_20970*), and the gene encoding a TIGR04197 family VII secretion system effector (*J8Y18_14985*). (ii) Disrupted genes at the insertion site were identified as genes encoding transcriptional regulatory factors, including the RNA polymerase sigma factor SigB (*J8Y18_05105*) and transcriptional regulatory factor genes *J8Y18_01585*, *J8Y18_16035*, and *J8Y18_09555*. (iii) Disrupted genes at the insertion site, not previously reported to be associated with biofilm synthesis, included genes such as nitrate/sulfite reductase (*J8Y18_07220*), cupin protein (*J8Y18_10090*), chitinase (*J8Y18_18390*), putative proteins (*J8Y18_18430*, *J8Y18_22905*, *J8Y18_08480*, *J8Y18_03975*), UDP-N-acetylglucosamine dehydrogenase *(J8Y18_19300*), coproporphyrinogen III oxidase *(J8Y18_04490*), acetyl lactate synthase small subunit (*J8Y18_07090*), DNA topoisomerase IV subunit A (*J8Y18_17815*), phosphoribosylamine-glycine ligase ThiC *(J8Y18_25845*), glycosyltransferase family I (*J8Y18_13760*), adenosine succinate hydrolase (*J8Y18_01690*), and UDP-N-acetylglucosamine lipid carrier transferase (*J8Y18_08880*) (Table 2).

The gene encoding adenosine succinate hydrolase, *J8Y18_01690*, plays a role in extracellular DNA production, making it crucial for biofilm formation in *B. cereus* [30]. Another transposon mutagenesis study identified the *purD* and *purH* genes involved in purine biosynthesis as essential for pellicle biofilm formation in environmental isolates of *B. cereus* [12]. In our study, the identification of the gene encoding adenosine succinate hydrolase, *J8Y18_01690*, confirms the importance of purine biosynthesis genes in biofilm formation in *B. cereus*. However, the fact that this mutant is also non-motile suggests that the presumed lack of extracellular DNA is not the sole reason for its biofilm defect.

The genes encoding nitrate/nitrite reductase (*J8Y18_07220*), UDP-N-acetylmuramate dehydrogenase *(J8Y18_19300*), and UDP-N-acetylgalactosamine lipid carrier transferase (*J8Y18_08880*) participate in sugar metabolism and redox reactions. Nitrate/nitrite reductases are predominantly intracellular enzymes capable of efficiently degrading nitrate, suggesting their involvement in *B. cereus* biofilm synthesis. UDP-N-acetylmuramate dehydrogenase oxidizes N-acetylmuramic acid, a component of peptidoglycan in bacterial cell walls, implying its role in *B. cereus* biofilm synthesis. UDP-N-acetylgalactosamine lipid carrier transferase is involved in extracellular polysaccharide reactions, a crucial component of biofilm formation [31]. Therefore, it is reasonable to hypothesize that this genomic region plays a role in the production of extracellular polysaccharides, influencing biofilm synthesis. Studies indicate the essential role of galactose metabolism in *B. subtilis* biofilm formation [32]. In our screening, the presence of this gene suggests a similar crucial role for galactose metabolism in *B. cereus* biofilm formation.

### 3.3. Identification of orbF, a Key Gene for Biofilm Synthesis in Bacillus cereus

**Figure 3:**
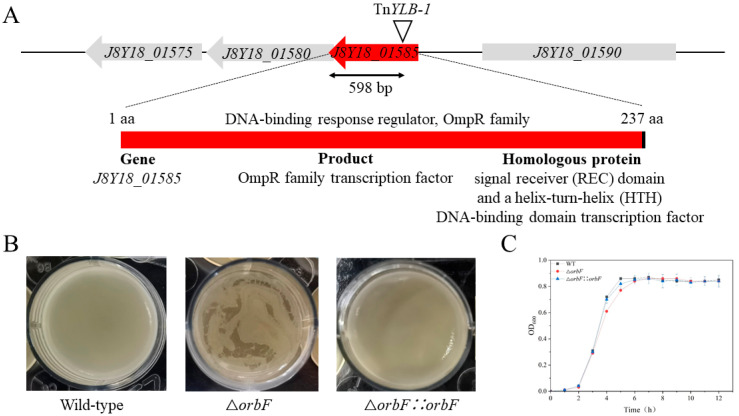
Discovery of the biofilm synthesis regulatory factor OrbF in BC1. (**A**) Relative positions of J8Y18_01575, J8Y18_01580, J8Y18_01585 and J8Y18_01590 in the B. cereus BC1 strain. The locus inserted by the transposon is marked with a triangle arrow. (**B**) The biofilm formation by strain BC1, strain BC1Δ*orbF* and strain BC1Δ*orbF::orbF*. (**C**) Growth pattern of strain BC1, strain BC1Δ*orbF* and strain BC1Δ*orbF::orbF* in Luria–Bertani (LB) medium.

Further investigation revealed that the disrupted gene in the mutant strain NJS-3-82 was identified as *J8Y18_01585*, encoding an OmpR family transcriptional regulatory factor. The insertion site was located between the 116th and 117th base pairs in the coding region of this gene (Figure 3A). Therefore, it is speculated that this transcription factor may be involved in the biofilm formation of BC1. Consequently, this gene was named *orbF* (**O**mpR family **r**egulator related to **b**iofilm **f**ormation). Subsequent in-frame deletion of the *J8Y18_01585* gene was performed, and the biofilm formation was compared among the wild type strain BC1, the mutant strain BC1Δ*orbF*, and the complemented strain BC1Δ*orbF::orbF*. The ability of BC1Δ*orbF* to form biofilm significantly decreased compared to the wild type strain BC1. The complemented strain restored the ability to synthesize biofilm (Figure 3B). Subsequently, we plotted the growth curves of the three strains. The results indicated that BC1Δ*orbF* exhibited slower growth during the logarithmic phase compared to the wild type strain BC1 and the complemented strain. However, once entering the stationary phase, the OD values of the three strains showed minimal differences (Figure 3C). Therefore, the possibility of a defect in biofilm synthesis due to growth conditions can be ruled out. These findings suggest that OrbF positively regulates the formation of biofilm in BC1.

### 3.4. OrbF Regulates BC1 Biofilm Synthesis, Flagellar Assembly, Bacterial Chemotaxis, and Community Sensing

**Figure 4:**
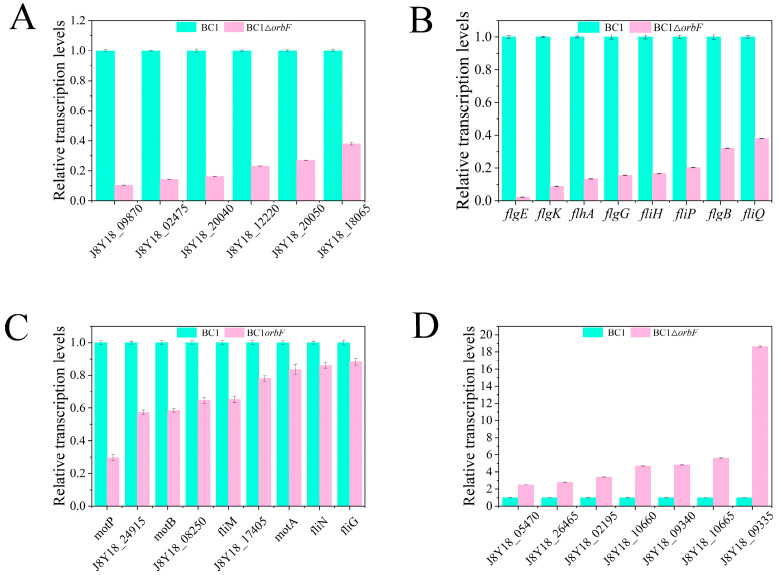
RT-qPCR analysis of (**A**) biofilm, (**B**) flagellar assembly, (**C**) bacterial chemotaxis, (**D**) quorum sensing-related gene transcription levels. The statistically significant differences were observed at a 5% confidence level of significance.

In this study, RT-qPCR was employed to analyze the transcription levels of genes related to quorum sensing, biofilm synthesis, flagellar assembly, and bacterial chemotaxis. The results indicated that, compared to the wild type strain, BC1Δ*orbF* showed significantly decreased expression of genes associated with biofilm synthesis, flagellar assembly, and bacterial chemotaxis (Figure 4A–C), while genes related to quorum sensing exhibited significantly increased expression (Figure 4D). These findings suggest that OrbF positively regulates the transcription of genes involved in BC1 biofilm synthesis, flagellar assembly, and bacterial chemotaxis, while negatively regulating the transcription of quorum sensing-related genes.

In this study, we identified 24 genes associated with the biofilm synthesis of *Bacillus cereus* BC1 (Table 2). However, only three encode proteins directly related to the flagellar complex. They are *J8Y18_08450*, encoding the flagellar basal rod protein FlgG; *J8Y18_22350*, the flagellar motor protein MotB; and *J8Y18_08360*, encoding the flagellar hook protein FlgE. Flagella are known to participate in biofilm formation in various bacterial species, and a fully functional flagellum requires dozens of genes. Studies by Houry et al. found that *fla* and *motAB* genes were implicated in the biofilm formation of *B. cereus* [33]. The remaining genes causing non-motile phenotypes include genes of unknown function, as well as previously unreported known functional genes affecting motility, such as *thiC* and *J8Y18_10090*, which will be discussed further in relation to the interplay between motility and biofilm formation.

The functionality of flagella, particularly functional flagellar systems, is crucial for the motility of *B. cereus*. Bacterial flagella consist of a supramolecular complex composed of the basal body, the hook, and the filament. RT-qPCR results indicate that in BC1Δ*orbF*, the transcription levels of relevant genes decreased by at least 65% (Figure 4B). Specifically, the transcription levels of genes related to basal body synthesis (*flgG*, *flgB*) decreased by 68.0% to 84.6%, and those related to hook synthesis (*flgE*, *flgK*) decreased by 91.3% to 97.8%. Additionally, the transcription levels of genes involved in flagellar biosynthesis proteins (*flhA*, *fliP*, *fliQ*) and genes related to flagellar assembly (*fliH*) also decreased by 62.0% to 96.7%.

**Figure 5:**
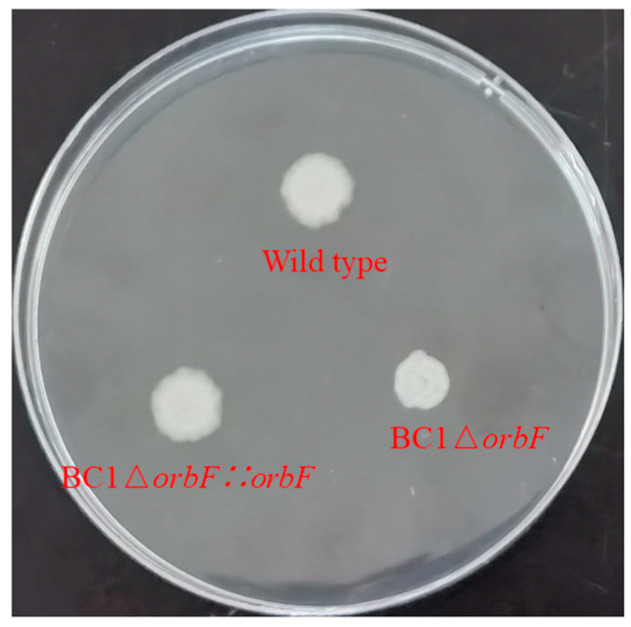
Swarming motility of wild type, BC1Δ*orbF*, and BC1Δ*orbF::orbF*.

Chemotaxis is one of the most fundamental physiological responses in bacteria, representing their ability to move. Although the *orbF* gene itself does not directly participate in motility, we observed impaired motility in BC1Δ*orbF* (Figure 5). Therefore, we speculate that swarming motility is a crucial factor for *B. cereus* BC1 in forming a pellicle biofilm. Consequently, RT-qPCR experiments were conducted on genes associated with bacterial chemotaxis. The results indicate that the transcription levels of genes related to the flagellar motor (*motP*, *motB*, *motA*), flagellar motor switch (*J8Y18_08250*, *fliM*, *fliN*, *fliG*), and methyl-accepting chemotaxis (*J8Y18_24915*, *J8Y18_17405*) decreased by 16.5% to 70.5%, 11.7% to 35.3%, and 22.0% to 42.6%, respectively (Figure 4C). This finding is consistent with a study by Houry and colleagues, who concluded that in static assays, *B. cereus* 407 strain forms a biofilm requiring motility, with functional flagella aiding bacteria in reaching the air–liquid interface [33].

In addition, *B. cereus* can regulate the expression of a series of target genes through quorum sensing and its interaction with transcription factors, thereby modulating microbial behaviors such as biofilm formation, acid resistance, and the regulation of virulence factors, leading to food spoilage and bacterial pathogenicity issues [14,15]. Interestingly, in the *orbF* mutant strain, we observed an upregulation of genes associated with quorum sensing, with their transcription levels increased by 1.48-fold to 17.6-fold. This suggests that OrbF may collaboratively regulate biofilm synthesis in *B. cereus* through quorum sensing. Well-studied quorum sensing systems in *Bacillus* species include Rap, NprR, and PlcR, identified as the initial members of the novel RNPP protein family. CodY regulates the expression of PlcR, indirectly controlling the expression of most known virulence factors in *B. cereus* [34,35]. Further research indicates that CodY controls the expression of virulence genes by regulating PapR [11], acting as a QS effector that activates PlcR [36]. Moreover, PlcR promotes the transcription of NprR, which positively regulates the transcription of kurstakin, a lipopeptide that promotes biofilm formation [37]. It is hypothesized that PlcR plays a crucial role in biofilm formation. PlcR has been found to inhibit the production of an unknown biosurfactant that contributes to biofilm formation under low-nutrient conditions [38]. However, the mechanism by which OrbF mediates quorum sensing to regulate biofilm synthesis in *B. cereus* remains to be explored.

### 3.5. Cloning and Expression of OrbF

**Figure 6:**
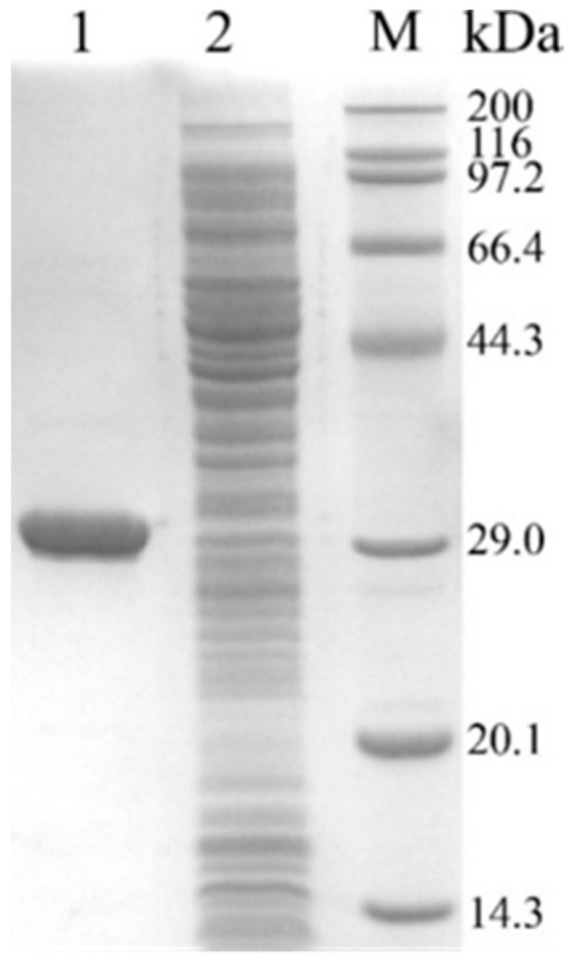
SDS-PAGE validation of transcription factor OrbF. M: Protein marker; Lane 1: Purified protein; Lane 2: *E. coli* BL21/pET-28a.

Using *B. cereus* BC1 genomic DNA as a template, the *orbF* gene was amplified by PCR with *orbF*-F and *orbF*-R as the upstream and downstream primers (Table S2), resulting in a 714 bp product (Figure S1). The gel-purified OrbF was ligated into the pMD18-T vector and sequenced. Blast analysis revealed 100% homology between its nucleotide sequence and the *orbF* gene sequence in the complete genomic sequence of *B. cereus*, encoding a 237-amino acid protein. The pMD18-T-*orbF* plasmid was digested with *Eco*R I and *Hin*d III, and the *orbF* fragment was gel-purified and ligated with pET-28a linearized with the same restriction enzymes to construct the recombinant vector pET-28a-orbF. This vector was then transformed into *E. coli* BL21(DE3) competent cells. Positive transformants were obtained, and the recombinant plasmid was extracted. Digestion of the recombinant plasmid pET-28a-*orbF* with *Eco*R I and *Hin*d III resulted in fragments of approximately 5369 bp and 714 bp, corresponding to the sizes of pET-28a and *orbF*, respectively. The successful construction of the recombinant plasmid pET-28a-*orbF* was confirmed (Figure S2). The *orbF* gene was cloned into the His-tagged expression vector pET-28a, creating the recombinant strain *E. coli* BL21/pET-28a-orbF. Induction and purification led to the isolation of the OrbF protein, showing a single band on SDS-PAGE gel electrophoresis, indicating the acquisition of a highly pure recombinant protein with a size of 28.4 kDa, consistent with the expected size (Figure 6).

### 3.6. OrbF Positively Regulates the Fla/Che Operon

**Figure 7:**
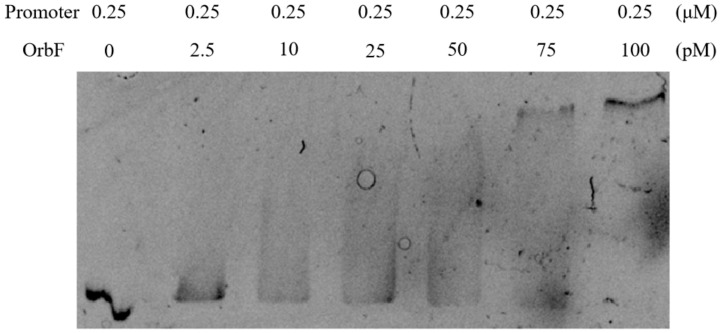
Binding of OrbF to the *fla*/*che* operon promoter.

Previous research has established that FlgE can influence the synthesis of the biofilm in *B. cereus* 892-1 [16] and, as demonstrated in this study through qPCR, the transcription level of *flgE* was downregulated in an *orbf*-deficient strain. However, the collaborative effect of OrbF and FlgE on the biofilm synthesis of *B. cereus* BC1 remains unverified. To address this, electrophoretic mobility shift assays (EMSA) were conducted to further analyze the impact of OrbF on the *fla*/*che* operon promoter (note that *flgE* is encoded within the long, 25-kb *fla*/*che* operon). As illustrated in Figure 7, the binding affinity increases with the quantity of OrbF protein, indicating OrbF’s interaction with the *fla*/*che* operon promoter. This binding affinity exhibits an upward gradient, even when the added biofilm synthesis gene cluster promoter concentration remains constant at 0.25 μM. The above result indicated that the regulation of OrbF in biofilm synthesis in *B. cereus* BC1 probably depended on FlgE.

## 4. Discussion

In food production, heat treatment is a commonly used sterilization method, and biofilm formation is a major mechanism through which foodborne microorganisms persist under pressure conditions, posing a significant concern in the fields of food nutrition and safety [39]. A biofilm is a structured community composed of bacterial cells of the same or different species, enhancing cell resistance to environmental stressors such as temperature changes, pH fluctuations, and desiccation, as well as resistance to antimicrobial agents [40,41,42,43]. While numerous studies have focused on the potential pathogenicity of microorganisms, little is known about how these genes are regulated under specific environmental conditions at the molecular level. Therefore, there is an urgent need to explore the regulatory and physiological mechanisms of foodborne microorganisms from a molecular perspective.

In this study, a TnYLB transposon library was successfully constructed, and mutant strains with defects in spore formation were screened from this library. Subsequent gene-level analysis and research were conducted using reverse PCR technology. Through the transposon library screening, 24 potential genes related to *B. cereus* biofilm synthesis were identified, including a novel transcriptional regulator, OrbF, RNA polymerase sigma factor SigB encoded by gene *J8Y18_05105*, and transcriptional regulators encoded by genes *J8Y18_01585*, *J8Y18_16035*, and *J8Y18_09555*, as well as nitrate/sulfate reductase encoded by gene *J8Y18_07220*. The phenotypic study of OrbF’s impact on *B. cereus* biofilm was conducted, and the potential molecular mechanism of OrbF’s regulation of biofilm synthesis was preliminarily elucidated.

Although this study has made certain progress in deciphering the mechanism of *B. cereus* biofilm formation, there are still pressing issues to be addressed: (i) How does OrbF regulate *B. cereus* biofilm formation at different growth stages? What are the specific binding sites? Therefore, there is an urgent need to explore the regulatory and physiological mechanisms of biofilm at the molecular level. (ii) In deciphering the mechanism of biofilm formation, due to the limitations of individual transcription factors’ effects, the signaling pathways remain at a “one-dimensional” level. Therefore, there is an urgent need for “multidimensional” research on the synergistic effects of multiple genes and conditions. (iii) We found that OrbF may mediate quorum sensing to regulate *B. cereus* biofilm synthesis, but the mechanism needs further investigation.

With the acquisition of the complete genome sequences of many microbial species, the focus of microbial attention and research has gradually shifted from structural genomics to functional genomics [44] and transcriptome [45]. Transposon random mutagenesis technology has become an effective tool for studying functional genomics (such as discovering new genes, cloning functional genes, and exploring new functions of known genes) due to its simple operation. Currently, the use of transposon random mutagenesis technology for *B. subtilis* research is relatively in depth, yielding genes related to sporulation, biofilm formation, and plant–microbe interactions [46,47,48]. As research on transposon technology deepens, work in this field for *B. cereus* is gradually expanding.

## Figures and Tables

**Table 1:** Comparison of transposition efficiency of TnYLB-1 under different transposition conditions.

Time	Transposition Efficiency (%)					
	40 °C	42 °C	44 °C	46 °C	48 °C	50 °C
4	21.46 ± 0.32 ^d^	24.68 ± 0.37 ^d^	29.35 ± 0.41 ^d^	34.76 ± 0.37 ^d^	12.34 ± 0.18 ^c^	11.98 ± 0.19 ^b^
6	36.77 ± 0.41 ^c^	37.41 ± 0.41 ^c^	41.26 ± 0.46 ^c^	48.23 ± 0.51 ^c^	19.78 ± 0.21 ^b^	29.16 ± 0.26 ^a^
8	42.59 ± 0.41 ^b^	44.16 ± 0.48 ^b^	57.26 ± 0.32 ^b^	71.17 ± 0.61 ^b^	28.93 ± 0.28 ^a^	-
10	52.76 ± 0.32 ^a^	59.39 ± 0.32 ^a^	76.47 ± 0.32 ^a^	89.77 ± 0.32 ^a^	-	-

Note: The transposition efficiency in the table represents the average of three replicate experiments. “±” indicates the standard deviation, and “-” signifies that there was no single colony growth on the Kan 50 µg/mL LB plate under that treatment condition, indicating no transposition of the TnYLB-1 transposon. Bold font highlights the optimal reaction conditions and their corresponding outcomes. The differences between groups are expressed by “a”, “b”, “c”, “d”, those lowercase letters in the table. *p* < 0.05.

**Table 2:** Genes disrupted in the *Bacillus cereus* BC1 genome.

Mutant	Gene (bp)	Product
NJS-1-11	*J8Y18_20970* (303)	type II secretion system protein
NJS-1-15	*J8Y18_08450* (774)	flagellar basal body rod protein FlgG
NJS-1-21	*J8Y18_14985* (276)	TIGR04197 family type VII secretion effector
NJS-1-36	*J8Y18_22350* (789)	flagellar motor protein MotB
NJS-1-57	*J8Y18_08360* (1221)	flagellar hook protein FlgE
NJS-1-89	*J8Y18_07220* (1623)	nitrite/sulfite reductase
NJS-2-24	*J8Y18_10090* (366)	cupin
NJS-2-38	*J8Y18_18390* (1083)	chitinase
NJS-2-59	*J8Y18_18430* (228)	hypothetical protein
NJS-2-72	*J8Y18_22905* (423)	hypothetical protein
NJS-3-02	*J8Y18_08480* (360)	hypothetical protein
NJS-3-46	*J8Y18_03975* (597)	hypothetical protein
NJS-3-78	*J8Y18_05105* (777)	RNA polymerase sigma factor SigB
NJS-3-82	*J8Y18_01585* (714)	response regulator transcription factor
NJS-3-93	*J8Y18_16035* (678)	response regulator transcription factor
NJS-4-11	*J8Y18_09555* (690)	response regulator transcription factor
NJS-4-21	*J8Y18_19300* (456)	UDP-N-acetylmuramate dehydrogenase
NJS-4-68	*J8Y18_04490* (1491)	coproporphyrinogen III oxidase
NJS-4-79	*J8Y18_07090* (510)	acetolactate synthase small subunit
NJS-5-10	*J8Y18_17815* (2424)	DNA topoisomerase IV subunit A
NJS-5-26	*J8Y18_25845* (1761)	phosphomethylpyrimidine synthase ThiC
NJS-5-26	*J8Y18_13760* (1194)	glycosyl transferase family 1
NJS-5-58	*J8Y18_01690* (1308)	adenylosuccinate lyase
NJS-5-73	*J8Y18_08880* (804)	UDP-galactose-lipid carrier transferase

## Data Availability

The original contributions presented in the study are included in the article and supplementary materials, further inquiries can be directed to the corresponding author.

## Supplementary Materials

The following supporting information can be downloaded at: https://www.mdpi.com/article/10.3390/foods13050638/s1, Figure S1: PCR amplification of *orbF* gene.; Figure S2: Enzyme analysis of pET-28a-*orbF*.; Table S1: Strains and plasmids used in this study; Table S2: Primers used in this study.

## References

[1] Pires S.M., Desta B.N., Mughini-Gras L., Mmbaga B.T., Fayemi O.E., Salvador E.M., Gobena T., Majowicz S.E., Hald T., Hoejskov P.S. (2021). Burden of foodborne diseases: Think global, act local. Curr. Opin. Food Sci..

[2] Oliveira M., Carvalho M., Teixeira P. (2023). Characterization of the Toxigenic Potential of *Bacillus cereus* sensu lato Isolated from Raw Berries and Their Products. Foods.

[3] Gao T., Ding Y., Wu Q., Wang J., Zhang J., Yu S., Yu P., Liu C., Kong L., Feng Z. (2018). Prevalence, Virulence Genes, Antimicrobial Susceptibility, and Genetic Diversity of *Bacillus cereus* Isolated from Pasteurized Milk in China. Front. Microbiol..

[4] Gupta P., Sarkar S., Das B., Bhattacharjee S., Tribedi P. (2016). Biofilm, pathogenesis and prevention--a journey to break the wall: A review. Arch. Microbiol..

[5] Liu C., Yu P., Yu S., Wang J., Guo H., Zhang Y., Zhang J., Liao X., Li C., Wu S. (2020). Assessment and molecular characterization of *Bacillus cereus* isolated from edible fungi in China. BMC Microbiol..

[6] Kong L., Yu S., Yuan X., Li C., Yu P., Wang J., Guo H., Wu S., Ye Q., Lei T. (2021). An Investigation on the Occurrence and Molecular Characterization of *Bacillus cereus* in Meat and Meat Products in China. Foodborne Pathog. Dis..

[7] Zhang Y., Chen M., Yu P., Yu S., Wang J., Guo H., Zhang J., Zhou H., Chen M., Zeng H. (2020). Prevalence, Virulence Feature, Antibiotic Resistance and MLST Typing of *Bacillus cereus* Isolated from Retail Aquatic Products in China. Front. Microbiol..

[8] Gram L., Ravn L., Rasch M., Bruhn J.B., Christensen A.B., Givskov M. (2002). Food spoilage—Interactions between food spoilage bacteria. Int. J. Food Microbiol..

[9] Cairns L.S., Hobley L., Stanley-Wall N.R. (2014). Biofilm formation by *Bacillus subtilis*: New insights into regulatory strategies and assembly mechanisms. Mol. Microbiol..

[10] Vlamakis H., Chai Y., Beauregard P., Losick R., Kolter R. (2013). Sticking together: Building a biofilm the *Bacillus subtilis* way. Nat. Rev. Microbiol..

[11] Slamti L., Lemy C., Henry C., Guillot A., Huillet E., Lereclus D. (2015). CodY Regulates the Activity of the Virulence Quorum Sensor PlcR by Controlling the Import of the Signaling Peptide PapR in *Bacillus thuringiensis*. Front. Microbiol..

[12] Yan F., Yu Y., Gozzi K., Chen Y., Guo J.H., Chai Y. (2017). Genome-Wide Investigation of Biofilm Formation in *Bacillus cereus*. Appl. Environ. Microbiol..

[13] Gastélum G., de la Torre M., Rocha J. (2020). Rap Protein Paralogs of *Bacillus thuringiensis*: A Multifunctional and Redundant Regulatory Repertoire for the Control of Collective Functions. J. Bacteriol..

[14] Slamti L., Perchat S., Huillet E., Lereclus D. (2014). Quorum sensing in *Bacillus thuringiensis* is required for completion of a full infectious cycle in the insect. Toxins.

[15] Verplaetse E., Slamti L., Gohar M., Lereclus D. (2015). Cell Differentiation in a *Bacillus thuringiensis* Population during Planktonic Growth, Biofilm Formation, and Host Infection. mBio.

[16] Li Y., Chen N., Wu Q., Liang X., Yuan X., Zhu Z., Zheng Y., Yu S., Chen M., Zhang J. (2022). A Flagella Hook Coding Gene flgE Positively Affects Biofilm Formation and Cereulide Production in Emetic *Bacillus cereus*. Front. Microbiol..

[17] Slack F.J., Serror P., Joyce E., Sonenshein A.L. (1995). A gene required for nutritional repression of the *Bacillus subtilis* dipeptide permease operon. Mol. Microbiol..

[18] Yan F., Yu Y., Wang L., Luo Y., Guo J.H., Chai Y. (2016). The comER Gene Plays an Important Role in Biofilm Formation and Sporulation in both *Bacillus subtilis* and *Bacillus cereus*. Front. Microbiol..

[19] Lin Y., Briandet R., Kovács Á.T. (2022). *Bacillus cereus* sensu lato biofilm formation and its ecological importance. Biofilm.

[20] Le Breton Y., Mohapatra N.P., Haldenwang W.G. (2006). In vivo random mutagenesis of *Bacillus subtilis* by use of TnYLB-1, a mariner-based transposon. Appl. Environ. Microbiol..

[21] Tagawa Y. (2014). Isolation and characterization of flagellar filaments from *Bacillus cereus* ATCC 14579. Antonie Van Leeuwenhoek.

[22] Chelliah R., Wei S., Park B.J., Kim S.H., Park D.S., Kim S.H., Hwan K.S., Oh D.H. (2017). Novel motB as a potential predictive tool for identification of *B. cereus*, *B. thuringiensis* and differentiation from other Bacillus species by triplex real-time PCR. Microb. Pathog..

[23] Sun Y., Wang L., Osire T., Fu W., Yi G., Yang S.-T., Yang T., Rao Z. (2021). Comparative transcriptome analysis reveals metabolic regulation of prodigiosin in *Serratia marcescens*. Syst. Microbiol. Biomanuf..

[24] Zhu Y., Ji F., Shang H., Zhu Q., Wang P., Xu C., Deng Y., Peng D., Ruan L., Sun M. (2011). Gene clusters located on two large plasmids determine spore crystal association (SCA) in *Bacillus thuringiensis* subsp. finitimus strain YBT-020. PLoS ONE.

[25] Arantes O., Lereclus D. (1991). Construction of cloning vectors for *Bacillus thuringiensis*. Gene.

[26] Okshevsky M., Louw M.G., Lamela E.O., Nilsson M., Tolker-Nielsen T., Meyer R.L. (2018). A transposon mutant library of *Bacillus cereus* ATCC 10987 reveals novel genes required for biofilm formation and implicates motility as an important factor for pellicle-biofilm formation. Microbiologyopen.

[27] Pan X., Sun C., Tang M., You J., Osire T., Zhao Y., Xu M., Zhang X., Shao M., Yang S. (2020). LysR-Type Transcriptional Regulator MetR Controls Prodigiosin Production, Methionine Biosynthesis, Cell Motility, H(2)O(2) Tolerance, Heat Tolerance, and Exopolysaccharide Synthesis in *Serratia marcescens*. Appl. Environ. Microbiol..

[28] Sun Y., Wang L., Osire T., Fu W., Yi G., Yang S.T., Yang T., Rao Z. (2021). Enhanced Prodigiosin Production in *Serratia marcescens* JNB5-1 by Introduction of a Polynucleotide Fragment into the pigN 3′ Untranslated Region and Disulfide Bonds into O-Methyl Transferase (PigF). Appl. Environ. Microbiol..

[29] Sun Y., Wang L., Pan X., Osire T., Fang H., Zhang H., Yang S.T., Yang T., Rao Z. (2020). Improved Prodigiosin Production by Relieving CpxR Temperature-Sensitive Inhibition. Front. Bioeng. Biotechnol..

[30] Vilain S., Pretorius J.M., Theron J., Brözel V.S. (2009). DNA as an adhesin: *Bacillus cereus* requires extracellular DNA to form biofilms. Appl. Environ. Microbiol..

[31] Bugert P., Geider K. (1995). Molecular analysis of the ams operon required for exopolysaccharide synthesis of *Erwinia amylovora*. Mol. Microbiol..

[32] Chai Y., Beauregard P.B., Vlamakis H., Losick R., Kolter R. (2012). Galactose metabolism plays a crucial role in biofilm formation by *Bacillus subtilis*. mBio.

[33] Houry A., Briandet R., Aymerich S., Gohar M. (2010). Involvement of motility and flagella in *Bacillus cereus* biofilm formation. Microbiology.

[34] Lindbäck T., Mols M., Basset C., Granum P.E., Kuipers O.P., Kovács Á.T. (2012). CodY, a pleiotropic regulator, influences multicellular behaviour and efficient production of virulence factors in *Bacillus cereus*. Environ. Microbiol..

[35] Gohar M., Faegri K., Perchat S., Ravnum S., Økstad O.A., Gominet M., Kolstø A.B., Lereclus D. (2008). The PlcR virulence regulon of *Bacillus cereus*. PLoS ONE.

[36] Slamti L., Lereclus D. (2002). A cell-cell signaling peptide activates the PlcR virulence regulon in bacteria of the *Bacillus cereus* group. Embo J..

[37] Gélis-Jeanvoine S., Canette A., Gohar M., Caradec T., Lemy C., Gominet M., Jacques P., Lereclus D., Slamti L. (2017). Genetic and functional analyses of krs, a locus encoding kurstakin, a lipopeptide produced by *Bacillus thuringiensis*. Res. Microbiol..

[38] Hsueh Y.H., Somers E.B., Lereclus D., Wong A.C. (2006). Biofilm formation by *Bacillus cereus* is influenced by PlcR, a pleiotropic regulator. Appl. Environ. Microbiol..

[39] Chmielewski R.A.N., Frank J.F. (2003). Biofilm Formation and Control in Food Processing Facilities. Compr. Rev. Food Sci. Food Saf..

[40] Flemming H.C., Wuertz S. (2019). Bacteria and archaea on Earth and their abundance in biofilms. Nat. Rev. Microbiol..

[41] Bisht K., Luecke A.R., Wakeman C.A. (2022). Temperature-specific adaptations and genetic requirements in a biofilm formed by *Pseudomonas aeruginosa*. Front. Microbiol..

[42] Li Y., Hu X., Ruan J., Arola D.D., Ji C., Weir M.D., Oates T.W., Chang X., Zhang K., Xu H.H.K. (2019). Bonding durability, antibacterial activity and biofilm pH of novel adhesive containing antibacterial monomer and nanoparticles of amorphous calcium phosphate. J. Dent..

[43] Dehbanipour R., Ghalavand Z. (2022). *Acinetobacter baumannii*: Pathogenesis, virulence factors, novel therapeutic options and mechanisms of resistance to antimicrobial agents with emphasis on tigecycline. J. Clin. Pharm. Ther..

[44] Caro-Astorga J., Frenzel E., Perkins J.R., Álvarez-Mena A., de Vicente A., Ranea J.A.G., Kuipers O.P., Romero D. (2020). Biofilm formation displays intrinsic offensive and defensive features of *Bacillus cereus*. NPJ Biofilms Microbiomes.

[45] Li S., Zhang B., Hu J., Zhong Y., Sun Y., Nie S. (2021). Utilization of four galactans by *Bacteroides thetaiotaomicron* A4 based on transcriptome. Food Front..

[46] Dempwolff F., Sanchez S., Kearns D.B. (2020). TnFLX: A Third-Generation mariner-Based Transposon System for *Bacillus subtilis*. Appl. Environ. Microbiol..

[47] Meeske A.J., Rodrigues C.D., Brady J., Lim H.C., Bernhardt T.G., Rudner D.Z. (2016). High-Throughput Genetic Screens Identify a Large and Diverse Collection of New Sporulation Genes in *Bacillus subtilis*. PLoS Biol..

[48] Wang P., Guo Q., Ma Y., Li S., Lu X., Zhang X., Ma P. (2015). DegQ regulates the production of fengycins and biofilm formation of the biocontrol agent *Bacillus subtilis* NCD-2. Microbiol. Res..

