# Supplementary material for: Investigating the Role of OrbF in Biofilm Biosynthesis and Regulation of Biofilm-Associated Genes in Bacillus cereus BC1
Source: Foods. 2024 Feb 20;13(5):638. doi: 10.3390/foods13050638 (PMC10931033; doi:10.3390/foods13050638)
Supplement: Supplementary file 1 [file foods-13-00638-s001.zip › foods-2795890-supplementary.pdf]

Supplementary Materials

Supplementary Figures

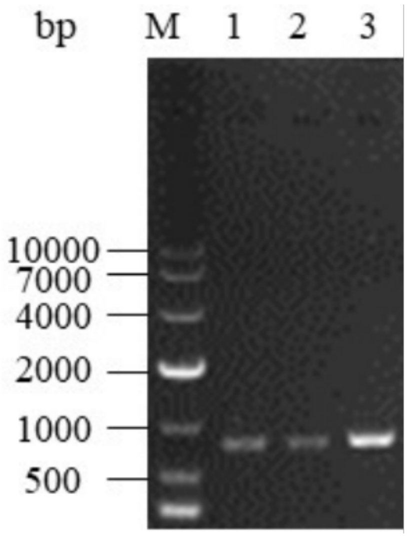

Figure S1. PCR amplification of *orbF* gene. M: 10000 DNA marker; lane 1 to lane 3: PCR product (*orbF*). The PCR product of *orbF* is 714 bp.

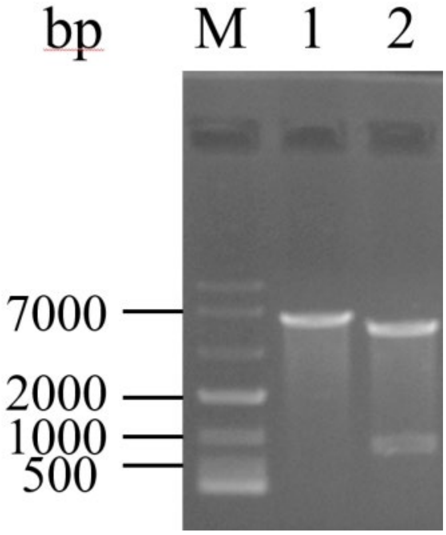

Figure S2. Enzyme analysis of pET-28a-*orbF*. M: 10000 DNA marker; lane 1: pET-28a-*orbF*; lane 2: pET-28a (the top strip, 5369bp) and *orbF* (the bottom strip, 714bp).

Supplementary Tables

Table S1 Strains and plasmids used in this study

Strains/Plasmids	Description	Source
Strains		
Bacillus cereus BC1	Isolated from pasteurized milk	Lab collection
E. coli DH5α	Storage plasmids	Invitrogen
E. coli BL21	Host for protein expression	Invitrogen
BC1△*orbF*	In-frame deletion of *orbF* in *Bacillus cereus* BC1	This work
BC1△*orbF* ∷*orbF*	Ectopic expression gene *orbF* in Δ*orbF*	This work
BL21/*orbF*	*E. coli* BL21 containing pET-28a- *orbF*, host for OrbF expression	This work
Plasmids		
pMarA-TnYLB-1	Construct transposon mutagenesis library	[20]
pHT304-TS	Knockout gene	[24]
pHT304	Complement gene	[25]
pHT304-TS-*orbF*	Knockout the gene *orbF*	This work
pHT304-*orbF*	Complement the gene *orbF*	This work
pET-28a	Expression of protein	Invitrogen
pET-28a-*orbF*	Expression of OrbF	This work
pMD19T	Ligation of promoter	Invitrogen
pMD19T-Promoter	Ligation of promoter	This work

Table S2 Primers used in this study

Primers	Sequence（5'-3'）	Note
oIPCR-F	GCTTGTAAATTCTATCATAATTG	Confirm transposon insertion site
oIPCR-R	AGGGAATCATTTGAAGGTTGG	
orbF-F	ctcgagtgcggccgcaagcttATGAAAGATATACGTATCCTTA	Amplification of *orbF* gene
orbF-R	atgggtcgcggatccgaattcTCAGTCTTCAAGCTTTTCACCTA	
orbF up-F	ACCATGATTACGCCAAGCTTTCAACTAGGTGCTTTAAGTTATTTG	Amplification of *orbF* up homologous arm, Knockout gene *orbF*
orbF up-R	CAGGTGAAGATATGAAAGATGTGAAAAGCTTGAAGACTGATAAAAC	
orbF down-F	TCAGTCTTCAAGCTTTTCACATCTTTCATATCTTCACCTGCTTC	Amplification of *orbF* down homologous arm, Knockout gene *orbF*
orbF down-R	CGACGTTGTAAAACGACGGCCAGTGAATTCAACTCTCCAATAAAGTTAGTTTGTC	
orbF-CP-F	ACCATGATTACGCCAAGCTTATGAAAGATATACGTATCCTTA	Amplification of *orbF* gene for complementation
orbF-CP-R	AAACGACGGCCAGTGAATTCTCAGTCTTCAAGCTTTTCACCTA	
09870-F	GTGTAGGAGCGGTTACTACT	RT-qPCR
09870-R	TTAATGCAGAAGGCCCCATC	RT-qPCR
02475-F	AATGGTTAGGTTTCTTTGCT	RT-qPCR
02475-R	CCTGAACCAATTGCACCTAA	RT-qPCR
20040-F	CACGCATTGCGTCAGTAATA	RT-qPCR
20040-R	ATGGTATTCATATTCACCCT	RT-qPCR
12220-F	TGATACACTAAAATACCTAA	RT-qPCR
12220-R	AAATATATGTTACCCCTCGA	RT-qPCR
20050-F	GAAACCAACGTTTGTAACGC	RT-qPCR

20050-R	AAACGTTAGCGATTGTTGCA	RT-qPCR
18065-F	GCACAACTTCAGCACCTTCT	RT-qPCR
18065-R	CATGGGGAATCAGATAGATT	RT-qPCR
fliE-F	TGTAGTTGAGGGGAAAAAGT	RT-qPCR
fliE-R	TACCCCTTTAGTTAGTAAAT	RT-qPCR
flgK-F	TGGCAAAAAATCCAGAACAA	RT-qPCR
flgK-R	TGCTTCCACTGTATCTAAGT	RT-qPCR
flhA-F	AGAGTTATTAGCGCATGAAG	RT-qPCR
flhA-R	AACGAGTGCGAATGGTAGAA	RT-qPCR
flgG-F	TGAGAAGAGTGAGATGTTAA	RT-qPCR
flgG-R	TGCTGCCTTAATAATGAACG	RT-qPCR
fliH-F	ATGAACAAATGGAACAGATG	RT-qPCR
fliH-R	TGCTAAATGAAATGATTGAT	RT-qPCR
fliP-F	AAGGACTTGGGGTAATGAAT	RT-qPCR
fliP-R	CACATCACTCTTCAGTTGCC	RT-qPCR
flgB-F	GATGAATAAAAGTAGTGCAT	RT-qPCR
flgB-R	ATTTACTGTCGGCAAGTGCA	RT-qPCR
fliQ-F	AAGTCTGATTGTCGTTATTA	RT-qPCR
fliQ-R	GAGGACAATACTCGCCATTT	RT-qPCR
motP-F	GTCTAAAAAGCACGGCTTGC	RT-qPCR

motP-R	ATCGTAACCGCTTAACATTA	RT-qPCR
24915-F	ACAACATTTTGAATCGATCC	RT-qPCR
24915-R	GCGGAAGAGGCGTCTAAGCA	RT-qPCR
motB-F	GAGGTGACGCTGAAACAAGT	RT-qPCR
motB-R	AGGGATTAATGGTGACAATA	RT-qPCR
08250-F	AGTGGAAACTGAGATTGTAG	RT-qPCR
08250-R	TAACTGGCTGATTTAAAAGC	RT-qPCR
fliM-F	GTCACAGACGTTATCAGCGC	RT-qPCR
fliM-R	CGGCATTTTCTCCACATATT	RT-qPCR
17405-F	ACATGACTAATTGTCTTAGT	RT-qPCR
17405-R	TACGAAAGAAATTGAAGAAG	RT-qPCR
motA-F	TGACCTAACTTTTCAATATC	RT-qPCR
motA-R	GCTATTTTTTCACAAGCTGG	RT-qPCR
fliN-F	TATTGATACTGTTTCAGATA	RT-qPCR
fliN-R	AACATCGCCAACTTTTAACT	RT-qPCR
fliG-F	ATATAATCTGATCGGTAAAT	RT-qPCR
fliG-R	CAAGCAATAACAGATGAATT	RT-qPCR
05470-F	CATTTACAATAAAGTAGCGG	RT-qPCR
05470-R	ATTCAGGTTTTAACATGAAT	RT-qPCR
26465-F	GTGGAAAGATAATCAAGCAC	RT-qPCR

26465-R	CGGCATCGGGATGGTCCTCG	RT-qPCR
02195-F	TATGACGTCACCGAAAGATT	RT-qPCR
02195-R	CTGTTTGTCCTAAGATGATT	RT-qPCR
10660-F	TGAATTATTAATGGCAGCTC	RT-qPCR
10660-R	TAGACTTTTGTTTCATGCCT	RT-qPCR
09340-F	GATAATTATTTACATTGCAC	RT-qPCR
09340-R	AGCTGCCATCCCTGCCCCTA	RT-qPCR
10665-F	TCGATTTATGTTGTCGTTGT	RT-qPCR
10665-R	GAATACAACGGATTTTTATA	RT-qPCR
09335-F	AGTGGGCTCTTGAGCGGAGG	RT-qPCR
09335-R	CTCCACCATTATCGGGGATA	RT-qPCR
